# Supplementary material for: Mutations in the efflux pump regulator MexZ shift tissue colonization by Pseudomonas aeruginosa to a state of antibiotic tolerance
Source: Nat Commun. 2024 Mar 22;15:2584. doi: 10.1038/s41467-024-46938-w (PMC10959964; doi:10.1038/s41467-024-46938-w)
Supplement: Supplementary file 1 — Supplementary information [file 41467_2024_46938_MOESM1_ESM.pdf]

Supplementary Table 1. Strains, plasmids and oligonucleotides used for this work.

**Bacterial strains**		
**Name**	**Description**	**Reference/origin**
PAO1	*P. aeruginosa* PAO1 wild type strain	[1]
*mexZ**	PAO1 strain with a C307T SNP in *mexZ* gene introduced by homologous recombination	This work.
*mexZ**Δ*lecA*	*mexZ** strain with deleted *lecA* by homologous recombination	This work
*mexZ**Δ*mexY*	*mexZ** strain with deleted *mexY* by homologous recombination	This work
*mexZ**OE*oprM*	*mexZ** strain overexpressing *oprM* gene	This work
Δ*mexB*	PAO1 strain with deleted *mexB* by homologous recombination	[2]
E. coli DH5α	Host strain used for the maintenance of cloning plasmids	Invitrogen
E. coli S17-1λ pir	Conjugative donor strain used for transferring plasmids to *P. aeruginosa* acceptor strains by conjugation assays	[3]

E. coli SM10(λ*pir*)/pTNS1	*E. coli* strain containing a pTNS1 plasmid used for four parental mating protocol for the introduction of the pUC18T-mini-Tn7T-Gm-Tp-*gfp* plasmid in *P. aeruginosa* by conjugation.	[4]
E. coli HB101/pRK2013	*E. coli* strain containing a pRK2013 plasmid used for four parental mating protocol for the introduction of the pUC18T-mini-Tn7T-Gm-Tp-*gfp* plasmid in *P. aeruginosa* by conjugation.	[4]
**Plasmids**		
**Name**	**Description**	**Reference/origin**
pUC18T-mini-Tn7T-Gm-Tp-*gfp*	Mobilizable TpR mini-Tn7 vector for GFP tagging bacteria. Gm^R	[4]
pFLP2	Vector used for excision of Gm^R marker in mini-Tn7 insertion strain by Flp recombinase. Cb^R	[4]
pEX18Ap	Conjugative plasmid used for deleting genes in *P. aeruginosa* by homologous recombination. Amp^R	[5]
pEX18Ap_*mexZ**	pEX18Ap plasmid containing the flanking DNA sequences of position 307 of *mexZ* gene with a C307T SNP used for introducing such genetic modification in *P. aeruginosa* PAO1 wild type strain. Amp^R	This work

pEX18Ap_Δ*lecA*	pEX18Ap plasmid containing the flanking DNA sequences of *lecA* gene used for deleting *lecA* in *P. aeruginosa mexZ** mutant. Amp^R	This work
pEX18Ap_Δ*mexY*	pEX18Ap plasmid containing the flanking DNA sequences of *mexY* gene used for deleting *mexY* in *P. aeruginosa mexZ** mutant. Amp^R	This work
pGEM-T Easy	Commercial plasmid used for cloning optimization of PCR products. Amp^R	Promega
pSEVA234	Plasmid used for overexpression of genes with a strong promoter controlled by IPTG. Km^R	[6]
pSEVA234*oprM*	Plasmid pSEVA234 used for overexpression of *oprM* gene with a strong promoter controlled by IPTG. Km^R	This work
**Oligonucleotides**		
**Name**	**Description**	**Sequence (5´-3´)**
EcoRI_*mexZ**Upstream_Fw	Used to amplify the upstream DNA flanking region for introduction of the C307T mutation in *mexZ* gene	CCCGAATTCAGCAGGAATAGGGCGACCA
*mexZ**Upstream_Rv		AGGATCTCCAGCACCCGCTA
*mexZ**Downstream_Fw	Used to amplify the downstream DNA flanking region for introduction of the C307T mutation in *mexZ* gene	CTGCGAGCCCGGTTCGGTGT
BamHI_*mexZ**Downtream_Rv		CCCGGATCCAGCGTGGAGATCGAAGGCAG

*mexZ**_comp_Fw	Used to check the introduction of C307T SNP in the *mexZ* gene	CGGTCTACGGCCACTACAAG
*mexZ**_comp_Rv		CGTTCGCACTTGAGGTAGAG
HindIII_*lecA*Upstream_Fw	Used to amplify the upstream DNA flanking region for deleting *lecA* gene	AAGCTTCCGGATGGCGAAATCGGTAG
*lecA*Upstream_Rv		AGACAAGTTATCAGGACTGATCCAAGCCATGATTGATCTC
*lecA*Downstream_Fw	Used to amplify the downstream DNA flanking region for deleting *lecA* gene	GAGATCAATCATGGCTTGGATCAGTCCTGATAACTTGTCT
HindIII_*lecA*Downstream_Rv		AAGCTTAATCGCAGCCAGCCTCGATG
Δ*lecA*_comp_Fw	Used to check the deletion of *lecA* gene	CTTCCTCGTTGCTGTGCTTT
Δ*lecA*_comp_Rv		GTGTTGAAGCAGTTCCCCG
HindIII_*mexY*Upstream_Fw	Used to amplify the upstream DNA flanking region for deleting *mexY* gene	AAGCTTAGGGTGTCGCCGACAAGGAC
*mexY*Upstream_Rv		GCTAGGGGCATCAGGCTTGCAACGAGCCATTCGTAGCGTT
*mexY*Downstream_Fw	Used to amplify the downstream DNA flanking region for deleting *mexY* gene	AACGCTACGAATGGCTCGTTGCAAGCCTGATGCCCCTAGC
HindIII_*mexY*Downstream_Rv		AAGCTTTCACCGATCTGTCGAGCCTC
Δ*mexY*_comp_Fw	Used to check the deletion of *mexY* gene	TTGGTGGAAGACGTGGAGG
Δ*mexY*_comp_Rv		TTGCCAGTCGTCCAGCTT
M13_Fw	Used to check the inserted DNA fragment into a pGEM-t Easy vector	CACGACGTTGTAAAACGAC
M13_Rv		GGATAACAATTTCACACAGG

AvrII_*oprM*_Fw	Used to amplify *oprM* by PCR introducing target sequences of restriction enzymes AvrII and HindIII	CCTAGGTCACACAGGAAACAGATGAAACGGTCCTTCCTTTC
HindIII_*oprM*_Rv		AAGCTTTCAAGCCTGGGGATCTTCCT
*rpsL*_Fw	Used to check DNA contamination in RNA samples	GCAAGCGCATGGTCGACAAGA
*rpsL*_Rv		CGCTGTGCTCTTGCAGGTTGTGA
*rplU*_Fw	Used to quantify *rplU* expression by RT-qPCR	CGCAGTGATTGTTACCGGTG
*rplU*_Rv		AGGCCTGAATGCCGGTGATC
*lecA*_Fw	Used to quantify *lecA* expression by RT-qPCR	ATAACGAAGCAGGGCAGGTA
*lecA*_Rv		TTGCCAATCTTCATGACCAG
*pqsD*_Fw	Used to quantify *pqsD* expression by RT-qPCR	CATGTGATCTGCCATCAACC
*pqsD*_Rv		AGCCGTAGGTCAGGACCAG

Supplementary Table 2. Minimal Inhibitory Concentrations (MICs) to tobramycin, ciprofloxacin and ceftazidime of the *Pseudomonas aeruginosa* strains used in this work, measured in Müller Hinton medium (MH), Pneumacult-ALI maintenance medium (ALI) and Synthetic Cystic Fibrosis sputum Medium (SCFM).

Strain	Medium	TOB MIC (µg/mL)	CIP MIC (µg/mL)	CAZ MIC (µg/mL)
PAO1	MH	1	0.2	2
	ALI	4	0.4	4
	SCFM	2	0.4	2
*mexZ**	MH	1.5	0.4	2
	ALI	6	0.8	4
	SCFM	4	0.8	2
*mexZ** Δ*lecA*	MH	1.5	0.4	2
	ALI	6	0.8	4
	SCFM	4	0.8	2
*mexZ** Δ*mexY*	MH	0.5	0.1	2
	ALI	2	0.2	4
	SCFM	0.5	0.2	2
Δ*mexB*	MH	1	0.1	0.5
	ALI	4	0.2	1
	SCFM	2	0.2	0.5

TOB: tobramycin; CIP: ciprofloxacin; CAZ: ceftazidime.

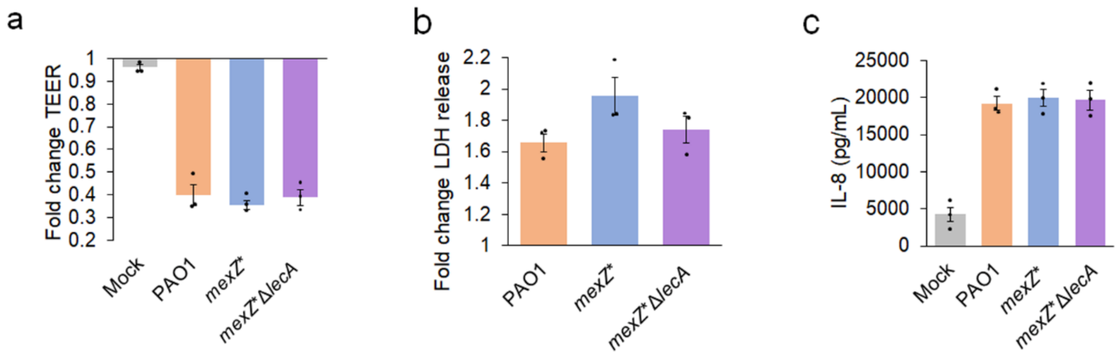

Supplementary Figure 1. Characterization of the effects of a mutation in *mexZ* for cell damage and immunogenicity in Air-Liquid Interface (ALI) BCi-NS1.1 airway infection models. a) Fold change of Transepithelial Electrical Resistance (TEER) (Ω·cm2) of mock BCi-NS1.1 ALI cell layers uninfected (grey) and after 14 hours of infection with PAO1 (orange), *mexZ** (blue) or *mexZ**Δ*lecA* (purple) respect to the TEER before starting the infection experiment. b) Fold change of LDH release of BCi-NS1.1 ALI cell layers after 14 hours of infection with PAO1 (orange), *mexZ** (blue) or *mexZ**Δ*lecA* (purple) respect to the mock uninfected control epithelia. c) IL-8 release (pg/mL) into the basolateral media indicating immunogenicity caused by the invading PAO1 (orange), *mexZ** (blue) or *mexZ**Δ*lecA* (purple) strains and basal levels of mock uninfected control BCi-NS1.1 epithelia (grey). Error bars indicate standard deviations relative to the mean of the results from three biological replicates for TEER measurements and three biological replicates with three technical replicates each for LDH and IL-8 measurements. No statistical significance was determined when comparing PAO1 and the mutant strains by performing a two-sided t-test assuming equal variances: TEER *mexZ** ($p = 0.44$), TEER *mexZ**Δ*lecA* ($p = 0.87$); LDH *mexZ** ($p = 0.088$), LDH *mexZ**Δ*lecA* ($p = 0.45$); IL-8 *mexZ** ($p = 0.64$), IL-8 *mexZ**Δ*lecA* ($p = 0.78$). Source data are provided as a Source Data file.

a

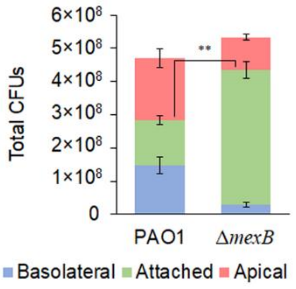

b

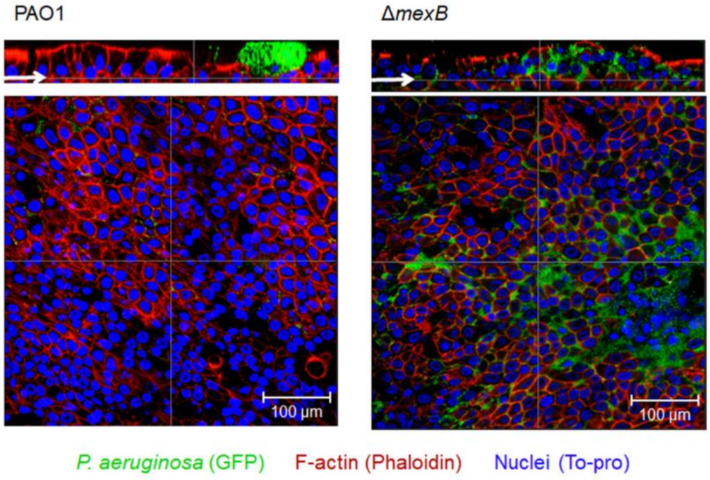

Supplementary Figure 2. Characterization of the effects of the loss of MexAB-OprM efflux pump for bacterial localization during colonization of Air-Liquid Interface (ALI) BCi-NS1.1 airway infection models. a) Colony Forming Units (CFU) of PAO1 and Δ*mexB* in the apical (red) and basolateral (blue) ALI compartments, and attached to the cell layer (green), after 14 hours of infection in fully differentiated BCi-NS1.1 cells. Error bars indicate standard deviations relative to the mean of the results from three biological replicates with three technical replicates each. Statistical significance was determined by two-sided t-test assuming equal variances for CFU measurements and indicated as ** ($p < 0.005$): attached CFUs PAO1 vs Δ*mexB* p-value = 0.00065. Source data are provided as a Source Data file b) Confocal images of the internal part of ALI cultures after 14 hours of infection with PAO1 or Δ*mexB P. aeruginosa* in green (GFP),

epithelial structure visualized by F-actin staining in red (Phalloidin) and nuclei in blue (To-pro), and their corresponding cross sections. Arrows in the cross section highlight the layer shown in the internal part image. Scale bar = 100 µm. The results shown were consistently obtained in 3 independent biological replicates of the experiment.

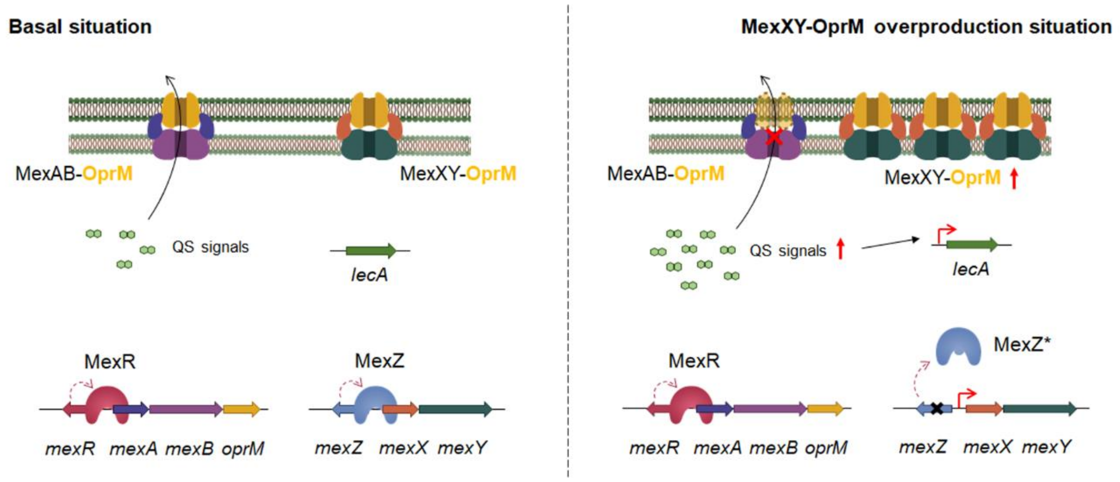

Supplementary Figure 3. Schematic representation of the mechanism underlying the infection phenotype associated with mutations in *mexZ*. In a basal situation, MexXY and MexAB efflux pumps share the porin OprM, and MexAB is involved in the extrusion of Quorum Sensing (QS) signals. A mutation in *mexZ* results in an increased production of the MexXY-OprM efflux pump, consequently decreasing the availability of OprM porin for the assembly of the MexAB-OprM efflux pump. This alteration leads to an increase in the intracellular concentration of QS signals typically extruded by MexAB-OprM. The disturbance in the QS signal levels triggers an upregulation of *lecA*, a gene encoding a lectin associated with *P. aeruginosa* invasiveness and its ability to attach to epithelial cells.

SUPPLEMENTARY REFERENCES

1. Stover, C. K. *et al.* Complete genome sequence of Pseudomonas aeruginosa PAO1, an opportunistic pathogen. *Nature* **406**, 959–964 (2000).

2. Laborda, P., Alcalde-Rico, M., Chini, A., Martínez, J. L. & Hernando-Amado, S. Discovery of inhibitors of Pseudomonas aeruginosa virulence through the search for natural-like compounds with a dual role as inducers and substrates of efflux pumps. *Environ. Microbiol.* **23**, 7396–7411 (2021).

3. Simon, R., O'Connell, M., Labes, M. & Puhler, A. Plasmid vectors for the genetic analysis and manipulation of rhizobia and other gram-negative bacteria. *Methods Enzym.* **118**, 640–659 (1986).

4. Choi, K. H. & Schweizer, H. P. mini-Tn7 insertion in bacteria with single attTn7 sites: Example Pseudomonas aeruginosa. *Nat. Protoc.* **1**, 153–161 (2006).

5. Hoang, T. T., Karkhoff-Schweizer, R. R., Kutchma, A. J. & Schweizer, H. P. A broad-host-range Flp-FRT recombination system for site-specific excision of chromosomally-located DNA sequences: application for isolation of unmarked Pseudomonas aeruginosa mutants. *Gene* **212**, 77–86 (1998).

6. Laborda, P., Martínez, J. L. & Hernando-Amado, S. Convergent phenotypic evolution towards fosfomycin collateral sensitivity of Pseudomonas aeruginosa antibiotic-resistant mutants. *Microb. Biotechnol.* **15**, 613–629 (2022).
