# Supplementary material for: Mutations in the efflux pump regulator MexZ shift tissue colonization by Pseudomonas aeruginosa to a state of antibiotic tolerance
Source: Nat Commun. 2024 Mar 22;15:2584. doi: 10.1038/s41467-024-46938-w (PMC10959964; doi:10.1038/s41467-024-46938-w)
Supplement: Supplementary file 3 — Description of Additional Supplementary Files [file 41467_2024_46938_MOESM3_ESM.pdf]

Description of Additional Supplementary Files:

Supplementary Dataset 1: Whole transcriptomic analysis of ΔmexB and mexZ* mutants. Number of Reads Per Kilobase Million (RPKM) of PAO1, ΔmexB and mexZ*, fold change and log2 fold change of the mutants respect to the wild type PAO1, as well as information about the genes are included in the Dataset. A graph showing the percentage of genes of each functional category (stablished by PseudoCap) presenting a log2 fold change ≤-1 or ≥1 is also included.

Supplementary Dataset 2: Expression changes in genes previously described to be regulated by Quorum Sensing in mexZ*and ΔmexB mutants. Log2 fold change of expression of genes previously related to Quorum Sensing of the mutants respect to the wild type PAO1 extracted from Supplementary Dataset 1 are included in this Dataset.
