# Supplementary material for: Mutations in the efflux pump regulator MexZ shift tissue colonization by Pseudomonas aeruginosa to a state of antibiotic tolerance
Source: Nat Commun. 2024 Mar 22;15:2584. doi: 10.1038/s41467-024-46938-w (PMC10959964; doi:10.1038/s41467-024-46938-w)
Supplement: Supplementary file 6 — Reporting Summary [file 41467_2024_46938_MOESM6_ESM.pdf]

nature portfolio

Corresponding author(s): Pablo Laborda

Last updated by author(s): Feb 15, 2024

Reporting Summary

Nature Portfolio wishes to improve the reproducibility of the work that we publish. This form provides structure for consistency and transparency in reporting. For further information on Nature Portfolio policies, see our Editorial Policies and the Editorial Policy Checklist.

Statistics

For all statistical analyses, confirm that the following items are present in the figure legend, table legend, main text, or Methods section.

n/a	Confirmed	
☐	☒	The exact sample size (*n*) for each experimental group/condition, given as a discrete number and unit of measurement
☐	☒	A statement on whether measurements were taken from distinct samples or whether the same sample was measured repeatedly
☐	☒	The statistical test(s) used AND whether they are one- or two-sided<br>*Only common tests should be described solely by name; describe more complex techniques in the Methods section.*
☒	☐	A description of all covariates tested
☒	☐	A description of any assumptions or corrections, such as tests of normality and adjustment for multiple comparisons
☐	☒	A full description of the statistical parameters including central tendency (e.g. means) or other basic estimates (e.g. regression coefficient) AND variation (e.g. standard deviation) or associated estimates of uncertainty (e.g. confidence intervals)
☐	☒	For null hypothesis testing, the test statistic (e.g. *F*, *t*, *r*) with confidence intervals, effect sizes, degrees of freedom and *P* value noted<br>*Give P values as exact values whenever suitable.*
☒	☐	For Bayesian analysis, information on the choice of priors and Markov chain Monte Carlo settings
☒	☐	For hierarchical and complex designs, identification of the appropriate level for tests and full reporting of outcomes
☒	☐	Estimates of effect sizes (e.g. Cohen's *d*, Pearson's *r*), indicating how they were calculated

Our web collection on statistics for biologists contains articles on many of the points above.

Software and code

Policy information about availability of computer code

Data collection	No custom software was used for data collection
Data analysis	The statistical analyses were performed using GraphPad Prism v9.5.0. For transcriptomic analysis, the numeric value of gene expression was normalized to Reads Per Kilobase of gene per million Mapped reads by using the CLC Genomics Workbench software 9.0 (Qiagen). Microscopy slides of infected cultures were analyzed using the LasX software 1.4.4.26810 (Leica) for infections on BCi-ExcelNS1.1 cells, and using the ZEN software 3.7.97.03000 (Zeiss) for infections on CF primary cells. The oligonucleotides used during this study were designed with Primer3 Input software 4.1.0. Graphs were generated by Excel v2401 and further finalized in PowerPoint v2401. Figure 4 was created by using Adove Illustrator v26.0.

For manuscripts utilizing custom algorithms or software that are central to the research but not yet described in published literature, software must be made available to editors and reviewers. We strongly encourage code deposition in a community repository (e.g. GitHub). See the Nature Portfolio guidelines for submitting code & software for further information.

Data

Policy information about availability of data

All manuscripts must include a data availability statement. This statement should provide the following information, where applicable:

- Accession codes, unique identifiers, or web links for publicly available datasets
- A description of any restrictions on data availability
- For clinical datasets or third party data, please ensure that the statement adheres to our policy

All data necessary for supporting the findings of this study are enclosed in this manuscript and its associated data indicated here. Raw data data generated in this study are provided in the Source Data file. Transcriptomic data included in this work are deposited in SRA database with accession code PRJNA990706 [https://www.ncbi.nlm.nih.gov/sra/PRJNA990706]. The PAO1 refence genome used during transcriptomic analysis can be found in GenBank database with the access code NC_002516.1 [https://www.ncbi.nlm.nih.gov/nuccore/NC_002516.1]. During transcriptomic analysis, genes were grouped in the functional classes stablished in PseudoCAP [https://pseudomonas.com/pseudocap]

Research involving human participants, their data, or biological material

Policy information about studies with human participants or human data. See also policy information about sex, gender (identity/presentation), and sexual orientation and race, ethnicity and racism.

Reporting on sex and gender	Sex or gender was not relevant for this study, since the investigation is focused on pulmonary infections by Pseudomonas aeruginosa which can affect people independently of those attributes.
Reporting on race, ethnicity, or other socially relevant groupings	Race, ethnicity or other socially relevant groupings were not relevant for this study, since the investigation is focused on pulmonary infections by Pseudomonas aeruginosa which can affect people independently of those attributes.
Population characteristics	A female patient with Cystic Fibrosis from the Copenhagen CF Center, born in 1998, homozygous with a ΔF508 mutation in the CFTR encoding gene.
Recruitment	The biological material was collected from a CF patient from the Copenhagen CF Center scheduled for extensive functional endoscopic sinus surgery during 2020 and 2023. Written informed consents were obtained prior surgery and biological material was obtained during the planned surgery.
Ethics oversight	Participants provided written informed consent. The project was approved by the local ethics committee of the Capital Region of Denmark (Region Hovedstaden) registration number H-20024750. All patients signed informed consent to participate in the study. Pseudoanonymisation of patients was carried out.

Note that full information on the approval of the study protocol must also be provided in the manuscript.

Field-specific reporting

Please select the one below that is the best fit for your research. If you are not sure, read the appropriate sections before making your selection.

☒ Life sciences ☐ Behavioural & social sciences ☐ Ecological, evolutionary & environmental sciences

For a reference copy of the document with all sections, see nature.com/documents/nr-reporting-summary-flat.pdf

Life sciences study design

All studies must disclose on these points even when the disclosure is negative.

Sample size	The sample size was not a relevant consideration for this study. The primary focus was on reconstructing a mutant in the gene mexZ and analysing its behaviour within a human lung infection system. Subsequent mutants were reconstructed with the aim of elucidating the molecular mechanisms underlying the observed infection phenotype.
Data exclusions	No data were excluded during this work.
Replication	The values obtained in this study were independently determined on at least three occasions, consistently yielding similar results. For infection experiments, three distinct transwells containing differentiated epithelia were employed to assess each mutant/condition, treating each transwell as a technical replicate of the infection. This experimental setup was replicated three times on different days, achieving what we defined as three biological replicates of the infection. Gene expression determinations were carried out with three biological replicates and three technical replicates. Minimal Inhibitory Concentrations were determined at least 3 times and the results were always consistent.
Randomization	Randomization was not relevant for this study, since not being a clinical trial. During this project, a mutant in the gene mexZ was specifically chosen to be reconstructed and analyse its behaviour in a human lung infection system. This was a hypothesis-driven research based on previous results that shown a paradox: the frequent selection of mutations in the gene mexZ despite its low contribution to antimicrobial resistance as measured in lab tests.
Blinding	As a general practice, we initially annotated the results without considering the plate or tube labels to minimize potential bias. Subsequently, we cross-referenced the annotations with the corresponding plate or tube labels.

Reporting for specific materials, systems and methods

We require information from authors about some types of materials, experimental systems and methods used in many studies. Here, indicate whether each material, system or method listed is relevant to your study. If you are not sure if a list item applies to your research, read the appropriate section before selecting a response.

Materials & experimental systems

n/a	Involved in the study
☒	☐ Antibodies
☐	☒ Eukaryotic cell lines
☒	☐ Palaeontology and archaeology
☒	☐ Animals and other organisms
☒	☐ Clinical data
☒	☐ Dual use research of concern
☒	☐ Plants

Methods

n/a	Involved in the study
☒	☐ ChIP-seq
☒	☐ Flow cytometry
☒	☐ MRI-based neuroimaging

Eukaryotic cell lines

Policy information about cell lines and Sex and Gender in Research

Cell line source(s)	The Basal Cell Immortalized Non-Smoker 1.1 (BCi-NS1.1, RRID:CVCL_T029) cell line was given by Professor Ronald G. Cristal (Weil Cornell Medical College, New York, USA).<br>The used cell line was previously described at: Walters MS, Gomi K, Ashbridge B, Moore MA, Arbelaez V, Heldrich J, Ding BS, Rafii S, Staudt MR, Crystal RG. Generation of a human airway epithelium derived basal cell line with multipotent differentiation capacity. Respir Res. 2013 Dec 3;14(1):135. doi: 10.1186/1465-9921-14-135.
Authentication	The cell line was morphologically (Bright Field, Immunofluorescence, flow cytometry) verified and tested for the CFTR expression by Western Blot.
Mycoplasma contamination	No mycoplasma contamination was detected during the course of this work.
Commonly misidentified lines<br>(See ICLAC register)	The Basal Cell Immortalized Non-Smoker 1.1 (BCi-NS1.1, RRID:CVCL_T029) cell line is not listed as a commonly misidentified line.

Plants

Seed stocks	*Report on the source of all seed stocks or other plant material used. If applicable, state the seed stock centre and catalogue number. If plant specimens were collected from the field, describe the collection location, date and sampling procedures.*
Novel plant genotypes	*Describe the methods by which all novel plant genotypes were produced. This includes those generated by transgenic approaches, gene editing, chemical/radiation-based mutagenesis and hybridization. For transgenic lines, describe the transformation method, the number of independent lines analyzed and the generation upon which experiments were performed. For gene-edited lines, describe the editor used, the endogenous sequence targeted for editing, the targeting guide RNA sequence (if applicable) and how the editor was applied.*
Authentication	*Describe any authentication procedures for each seed stock used or novel genotype generated. Describe any experiments used to assess the effect of a mutation and, where applicable, how potential secondary effects (e.g. second site T-DNA insertions, mosiacism, off-target gene editing) were examined.*
